# An Uncommon Journey of Melanoma In Situ: A Case Report of Metastasis to the Ureter and Porta Hepatis With Positive Urine Cytology

**DOI:** 10.7759/cureus.103152

**Published:** 2026-02-07

**Authors:** Jewel John, Amruta Patil, Allwyn John Karikunnel, Ashlin Z Thomas

**Affiliations:** 1 Department of Internal Medicine, Milton Keynes University Hospital, Milton Keynes, GBR; 2 Department of Pathology, Milton Keynes University Hospital, Milton Keynes, GBR; 3 Department of Emergency Medicine, Milton Keynes University Hospital, Milton Keynes, GBR; 4 Department of Critical Care Medicine, Believers Church Medical College Hospital, Thiruvalla, IND

**Keywords:** melanoma in-situ, metastatic melanoma, porta hepatis, ureter, urine cytology

## Abstract

Melanoma in situ is typically associated with an excellent prognosis following complete excision, with metastatic spread considered exceedingly rare. We present an exceptionally rare case of a 77-year-old woman with a history of completely excised melanoma in situ diagnosed four years back, who developed obstructive uropathy. Imaging revealed masses involving the ureter, porta hepatis region, and spine. Histopathological analysis of samples obtained via endoscopic ultrasound (EUS)-guided porta hepatis biopsy and ureteral tumor debris collected during cystoscopy confirmed Melan-A and S-100-positive metastatic malignant melanoma. No evidence of a new or recurrent primary cutaneous lesion was identified. This case highlights the rare potential for late and distant metastasis from melanoma in situ, including to atypical sites such as the ureter. It emphasizes the necessity of maintaining careful clinical monitoring throughout long-term follow-up.

## Introduction

Melanoma in situ (MIS) is traditionally considered a non-invasive form of melanoma confined to the epidermis, with a negligible risk of metastasis. However, rare instances of MIS exhibiting occult invasive components have been reported, leading to distant metastases. This case report presents an unusual progression of MIS, which, after a prolonged period of quiescence, metastasized to the ureter, porta hepatis, and spine. Her urine cytology also revealed the presence of tumor cells morphologically similar to the ureter biopsy. Metastatic melanoma in the porta hepatis can present with non-specific symptoms, often leading to diagnostic challenges. Similarly, spinal and ureter metastases from melanoma are relatively uncommon but can occur, particularly in advanced stages of the disease.

Although metastatic tumor deposits can be identified with imaging modalities like computed tomography (CT) and magnetic resonance imaging (MRI), the precise diagnosis of metastatic melanoma can only be clarified by immunohistochemical staining for markers like HMB-45 (human melanoma black-45), Melan-A (melanocytic differentiation marker), and S-100 (a family of calcium-binding proteins), which can aid in distinguishing melanoma from other malignancies [1].

Metastatic malignant melanoma is often associated with poor prognosis and a short survival cycle. The management of metastatic melanoma has evolved with the advent of immunotherapy, significantly improving survival outcomes. Agents targeting immune checkpoints, such as PD-1 (programmed cell death-1) inhibitors, have shown efficacy in treating metastatic melanoma, including cases with liver and spinal involvement.

After an extensive literature search, we found no prior reports of MIS with widespread metastasis, making this the first documented case. This case underscores the importance of vigilant long-term follow-up in patients with a history of MIS, as late metastasis can occur even after an extended period without recurrence. It also highlights the need for a multidisciplinary approach in diagnosing and managing metastatic melanoma, incorporating advanced imaging techniques, histopathological evaluation, and contemporary therapeutic strategies.

## Case presentation

**Figure 1:**
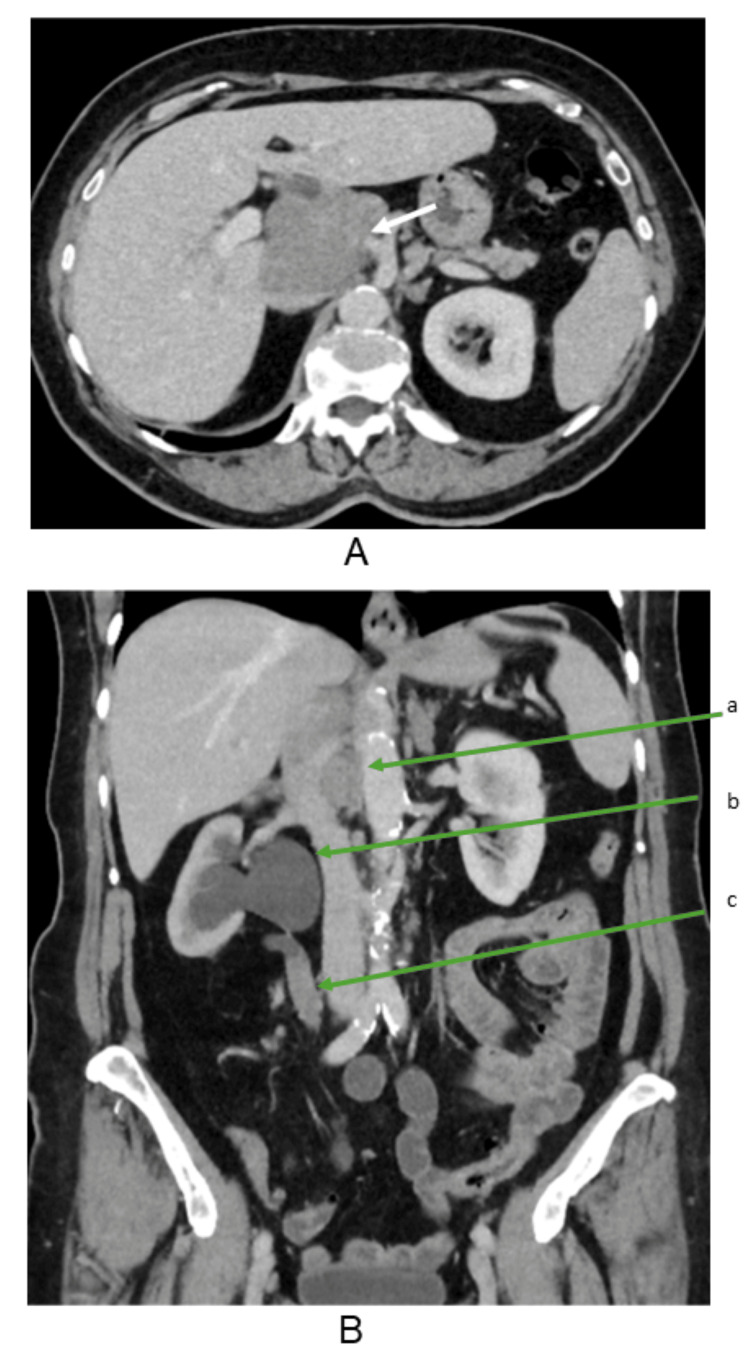
CT chest, abdomen, and pelvis (A) Axial CT image showing enlarged periportal lymph nodes (arrow). (B) Coronal CT image demonstrating (a) periportal lymph nodes, (b) right hydronephrosis, and (c) right ureteric soft tissue thickening CT: computed tomography

**Figure 2:**
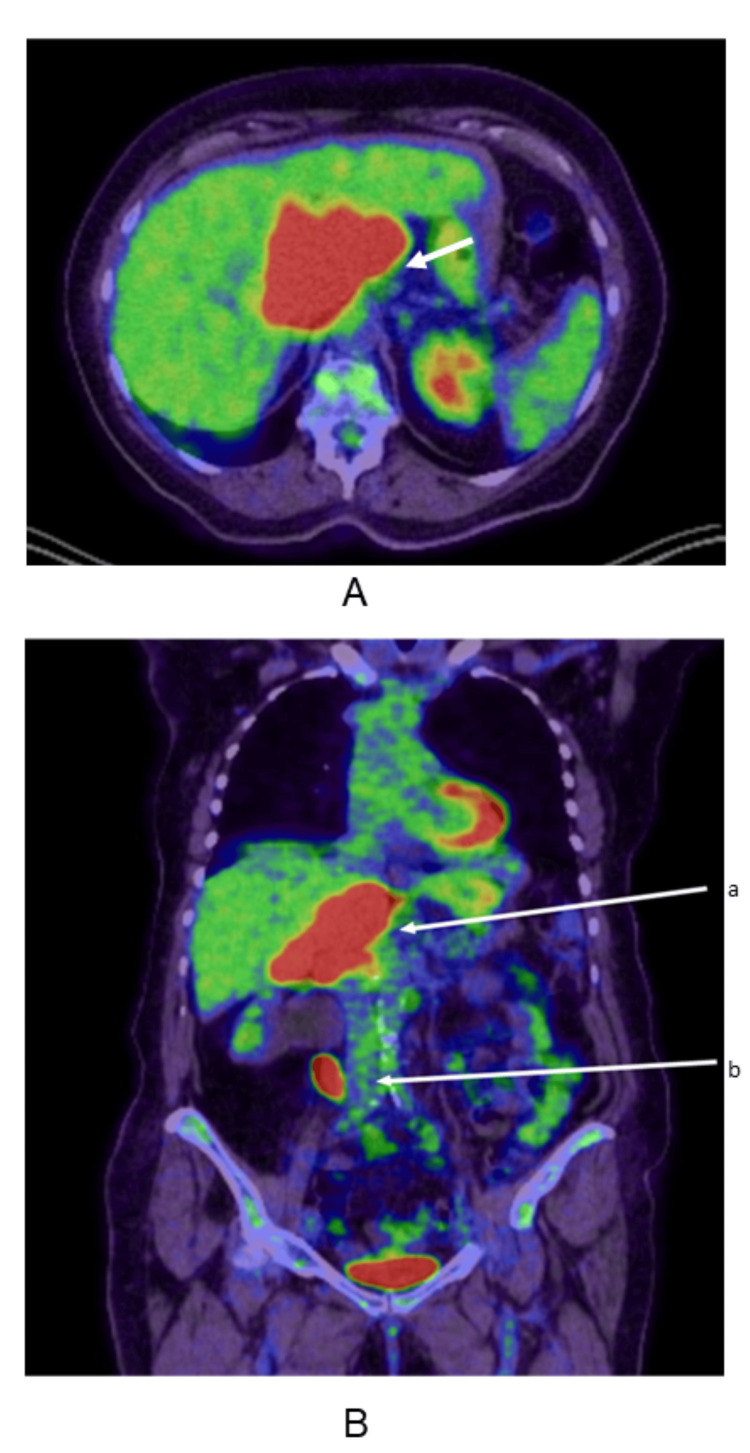
PET-CT scan (A) Axial PET-CT image showing increased FDG avidity in the periportal region(arrow). (B) Coronal PET-CT image depicting (a) FDG-avid periportal lymph nodes and (b) right ureteric soft tissue thickening PET-CT: positron emission tomography-computed tomography; FDG: fluorodeoxyglucose

A 77-year-old woman with biopsy-confirmed MIS on the right mid-back excised four years ago and a superficial micronodular basal cell carcinoma (BCC) excised from the upper back 15 years ago presented with visible hematuria, intermittent right-sided lower abdominal pain, fluctuating bowel habits (alternating between diarrhea and constipation), and increasing fatigue and nausea for four to five months unresponsive to multiple courses of antibiotics. Initial blood tests revealed a mildly cholestatic pattern on liver function tests. Tumor markers showed a moderately raised CA 19-9 (68 U/mL) with elevated fecal calprotectin (2,000 µg/g). She also tested positive for ANA (antinuclear antibody) and AMA (antimitochondrial antibody), consistent with her diagnosis of primary biliary cholangitis (Figures 1, 2).

A contrast-enhanced CT scan confirmed an 11.3 cm lobulated porta hepatis mass abutting the pancreatic head and duodenum. Another 12 mm arterially enhancing lesion in segment VIII of the liver was suspicious for metastasis. Additionally, an enhancing 3.7 cm right mid-ureteric mass was identified as the cause of hydronephrosis. MRI with liver-specific contrast demonstrated a periportal nodal mass with heterogeneous enhancement encasing the portal vein without obstruction, along with severe right hydronephrosis due to ureteric mural thickening.

Given the atypical location and absence of intrahepatic biliary dilatation, differential diagnoses included dual primary malignancies-a primary urothelial carcinoma of the ureter and a separate porta hepatis lesion, possibly metastatic or lymphoproliferative in origin. PET-CT (positron emission tomography-CT) showed metabolically active uptake at the porta hepatis, right mid-ureter, and T9 vertebral level, suggesting possible osseous metastasis.

**Figure 3:**
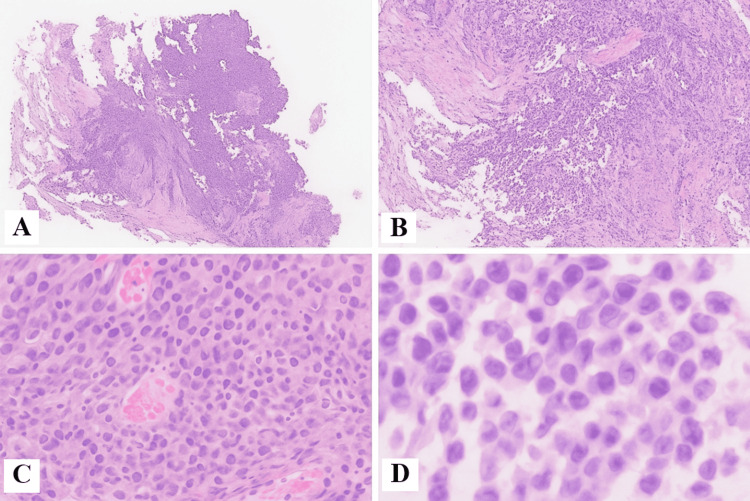
Histopathology of ureteric biopsy (H&E stain) (A and B) Malignant tumor with focal papillary architecture and necrosis (H&E 4x and 10x); (C and D) malignant cells with epithelioid morphology, rare intracytoplasmic melanin pigment, and prominent nucleoli (H&E 20x and 40x)

**Figure 4:**
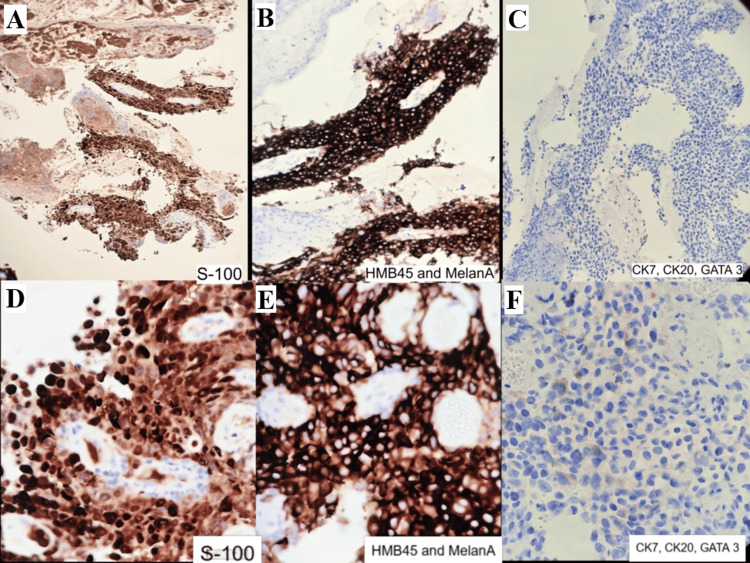
IHC staining of ureteric lesion (A-F) IHC on ureteric biopsy with tumor cells highlighted by Melan-A, S-100, and HMB45 and negative for CK7, CK20, and GATA3 IHC: immunohistochemistry

She underwent an EUS (endoscopic ultrasound)-guided biopsy of the porta hepatis mass outside our hospital, which confirmed metastatic melanoma on both morphology and immunohistochemistry (IHC). The ureteric biopsy done in our hospital on histopathology revealed atypical epithelioid cells with melanin pigment arranged in nests with focal necrosis. IHC showed tumor cells positive for S-100 and Melan-A and negative for CK7 (cytokeratin 7), CK20 (cytokeratin 20), and GATA3 (GATA-binding protein 3), confirming metastatic malignant melanoma (Figures 3, 4).

**Figure 5:**
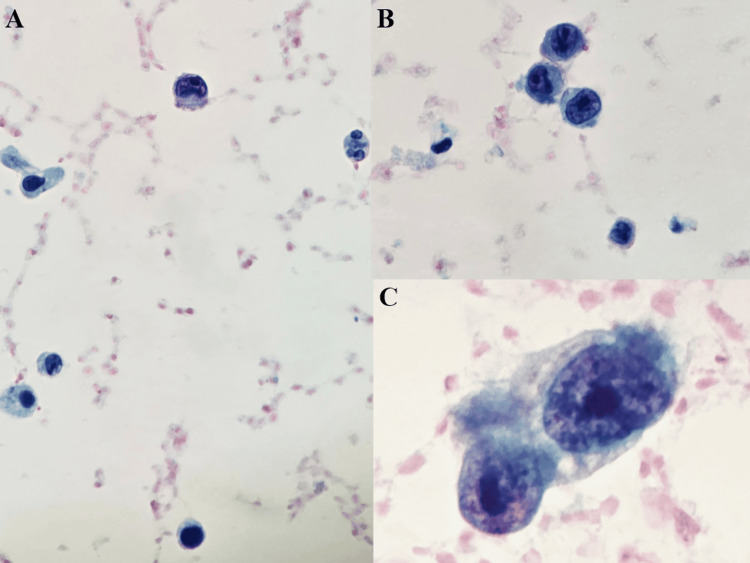
Urine cytology (A-C) Urine cytology with tumor cells showing a high N:C ratio, high-grade nuclear features with prominent nucleoli (C), and background inflammatory cells (Pap, 20x, 40x)

Her urine cytology also reveals the presence of highly atypical cells with nuclear atypia, prominent nucleoli, and high-grade features, similar to the morphology of cells in the ureter biopsy. Cystoscopy revealed no bladder abnormality. Due to tortuous anatomy, flexible ureteroscopy was limited; however, necrotic tumor debris was retrieved for histopathology, showing no urothelial carcinoma. The cells displayed pleomorphic nuclei with eosinophilic nucleoli, intracytoplasmic pigment, and an identical immunophenotype to the porta hepatis lesion, confirming metastatic melanoma (Figure 5).

On dermatology review, there were no new suspicious skin lesions or locoregional recurrence. Given the complex histopathology of the original melanoma and current findings, the patient's earlier lesion was likely to have unrecognized invasive or metastatic potential. She was diagnosed with disseminated metastatic melanoma involving the porta hepatis, right ureter, and T9 vertebra and urgently referred to Oncology to initiate systemic immunotherapy with pembrolizumab.

## Discussion

Malignant melanoma constitutes less than 5% of all skin malignancies [2]. It can metastasize in early stages, with the most common sites including the lymphatic system, lungs, brain, and liver. Approximately 0.2%-1% of melanoma cases metastasize to the genitourinary system, with obstructive uropathy being even rarer [3,4]. The common ureter malignancies are urothelial carcinoma (transitional cell carcinoma), squamous cell carcinoma, and adenocarcinoma. Common tumors metastasizing to the ureter include cancers of the stomach, breast, prostate, lung, and colon. Lymphomas can also involve the ureter through direct extension or systemic disease.

Melanoma is notorious for its propensity to metastasize to multiple organs and remains one of the most aggressive skin cancers. However, metastasis to the urinary tract is rare. Such involvement can cause obstructive uropathy, characterized by a blockage in the urinary pathway that leads to swelling and dilation of the urinary structures above the obstruction [4,5]. Less than 1% of melanomas metastasize to the genitourinary system, of which the majority involve the bladder; metastasis to the ureter is even more rare [6].

Melanoma is often described in the literature as the “great mimicker” because of its varied morphology and histological features, leading to a broad differential diagnosis, including poorly differentiated carcinomas, lymphomas (especially small cell or lymphoblastic types), sarcomas, plasmacytomas, neuroendocrine tumors, and other small round cell tumors [7]. IHC for the typical markers helps rule out other differentials.

In this case, the ureteral lesion initially raised suspicion for primary urothelial carcinoma due to obstruction, hematuria, and gross appearance. The lesion exhibited papillary architecture typical of urothelial tumors. However, atypical cell morphology and intracytoplasmic pigment prompted consideration of melanoma. This unusual combination underscored the importance of morphological evaluation and consideration of rare metastases.

IHC plays a crucial role in confirming metastatic melanoma diagnosis, especially when the primary tumor is unknown or morphology overlaps with other malignancies. Markers such as Pmel-17 (premelanosome protein)/gp100 (glycoprotein 100), MART-1/Melan-A, tyrosinase (melanosome-derived), S100B protein (cytoplasmic), MITF (microphthalmia-associated transcription factor), and SOX10 (nuclear) are used. Positive expression of Pmel-17/gp100 and MART-1/Melan-A supports melanoma diagnosis [1,8].

Detection of melanoma cells in urine is rare but can be an essential diagnostic tool for genitourinary melanoma. Large atypical, pigmented cells with eccentric nuclei and prominent nucleoli strongly suggest melanoma.

No documented case reports exist showing true MIS metastasizing to the ureter, as in situ tumors are generally non-invasive and incapable of metastasis. In this patient, no recurrence was observed at the original excision site. However, four years later, she presented with metastasis to the porta hepatis, ureter, and spine, which is extremely difficult to explain given the absence of any known primary melanoma elsewhere on the skin. In such a scenario, it becomes challenging to determine whether these lesions represent metastasis from the previous in situ lesion or a new primary melanoma. Advanced molecular studies like next-generation sequencing (NGS) may help clarify this distinction by comparing the genetic profiles of previous and current lesions. For instance, a study published in JAMA Dermatology found that approximately one-third of lesions diagnosed as MIS contained occult microinvasion detectable only through deeper tissue sectioning or IHC staining [9].

Among the few reported cases of metastatic MIS, only three confirmed true MIS (melanoma confined to epidermis without dermal invasion). Two showed regression, while other cases revealed invasive components on further sectioning or staining [10]. In the present case, there was no evidence of regression on the primary biopsy from the lesion on the back.

Once melanoma metastasizes to distant organs like the liver and spine, the prognosis is poor. The five-year survival rate for stage IV melanoma, including distant metastasis, is approximately 32%. Patients with liver metastasis have a grim prognosis, with five-year survival rates between 5% and 19%. Spinal metastases carry similarly poor outcomes. A study by Letho and Birendra reported a median survival of 4% with a two-year survival rate of 0.2% and a five-year survival rate below 1% for patients with spinal metastasis from melanoma [11].

Additional literature highlights that metastatic melanoma involving the genitourinary tract is rare, with ureteral involvement being particularly uncommon [4,6]. These studies emphasize the diagnostic challenges due to non-specific symptoms and the need for IHC confirmation [12].

A study published in JAMA Network Open analyzed long-term survival in patients with advanced melanoma treated with immune checkpoint inhibitors (ICIs) and reported a five-year overall survival rate of approximately 36%, indicating durable responses in a subset of patients [9]. These findings underscore the potential of immunotherapy to offer long-term survival benefits, even in patients with metastatic melanoma involving challenging sites like the porta hepatis and ureter. However, the rarity of MIS metastasizing to these sites necessitates further research to understand the underlying mechanisms and optimize treatment strategies. No explicit guidelines exist for managing genitourinary metastasis of melanoma, but surgical resection has been used when possible, in conjunction with immunotherapy [4,6].

## Conclusions

This case shows what is generally considered impossible-the rare potential for MIS to give rise to late, distant metastasis involving atypical sites such as the porta hepatis, right ureter, and T9 vertebra. It underscores the importance of considering metastatic melanoma in the differential diagnosis of ureteral masses, especially in patients with a history of melanoma, even in situ after years of excision. Careful histopathological evaluation with appropriate immunohistochemical markers is vital for accurate diagnosis. The finding had severe implications for the patient and raised the question of whether to intensify follow-up or extend the surgery in patients with MIS.
